# Journal of pharmaceutical health care and sciences

**DOI:** 10.1186/s40780-014-0010-3

**Published:** 2015-01-28

**Authors:** Toshiya Katsura

**Affiliations:** College of Pharmaceutical Sciences, Ritsumeikan University, 1-1-1 Noji-higashi, Kusatsu, Shiga 525-8577 Japan

## Editorial

We are proud to announce the launch of a new journal, *Journal of Pharmaceutical Health Care and Sciences (JPHCS)* [1]. *JPHCS* is an open access, peer-reviewed online journal that publishes research in all aspects of clinical pharmacy as well as clinical and experimental pharmacotherapy. The aim of the journal is to offer an open access forum for the publication of scientifically rigorous articles focusing on all fields of pharmaceutical health care and sciences.

*JPHCS* is the official journal of the Japanese Society of Pharmaceutical Health Care and Sciences (JSPHCS), which is founded in 1990. It aims to strengthen the foundation of Pharmaceutical Sciences through the exchange of knowledge between society members and relative societies for the benefit of public health and professional education. As of October 2014, the JSPHCS has over ten thousand members, including pharmacists, pharmaceutical researchers, and pharmaceutical company staff.

A new English journal in pharmaceutical health care and sciences is needed because there is a lack of journals in this area, particularly in the Asia-Pacific region. More importantly, there are few open access journals in this field worldwide. As an open access journal [2], all articles are freely, permanently and universally accessible online at no cost and it will facilitate greater dissemination in the world not restricted to Asia. Therefore, *JPHCS* has the potential to reach a much larger set of readers than any subscription-based journals. In addition, articles will be published as soon as they are accepted, and will be archived in PubMed Central [3] and other freely accessible full-text repositories. Authors will continue to hold copyright for their work and grant anyone the right to reproduce and disseminate the article, provided that it is correctly cited and no errors are introduced. We hope that the open access nature of *JPHCS* will help to fulfill the aim of the Society and to promote the exchange of ideas internationally in this and related fields.

*JPHCS* will publish articles relevant to clinical pharmacy as well as clinical and experimental pharmacotherapy including outcome research on pharmacotherapy and pharmaceutical health care, rational and appropriate pharmacotherapy in relation to clinical and/or experimental indices, pharmacoepidemiology, pharmacoeconomics, drug informatics, pharmacy services research, medication management, and other aspects of pharmaceutical health care and sciences. *JPHCS* welcomes papers in the following categories: Reviews (by invitation only), Research articles, Case reports and Short reports. Each article type published by *JPHCS* follows a specific format, as detailed in the corresponding instructions for authors [4].

Manuscripts submitted to *JPHCS* will be reviewed independently by at least two experts in the field. We are interested in publishing articles of the highest quality and novelty. We believe that *JPHCS* will become an important journal by providing opportunities for communication among the scientists of this field in the world, and we are looking forward to receiving your submissions.
